# Dichloroacetate improves systemic energy balance and feeding behavior during sepsis

**DOI:** 10.1172/jci.insight.153944

**Published:** 2022-06-22

**Authors:** Tae Seok Oh, Manal Zabalawi, Shalini Jain, David Long, Peter W. Stacpoole, Charles E. McCall, Matthew A. Quinn

**Affiliations:** 1 Department of Pathology, Section on Comparative Medicine, and; 2 Department of Internal Medicine, Section of Molecular Medicine, Wake Forest School of Medicine, Winston-Salem, North Carolina, USA.; 3 Division of Endocrinology, Diabetes and Metabolism, Department of Medicine and Department of Biochemistry and Molecular Biology, University of Florida College of Medicine, Gainesville, Florida, USA.

**Keywords:** Immunology, Bacterial infections

## Abstract

Sepsis is a life-threatening organ dysfunction caused by dysregulated host response to an infection. The metabolic aberrations associated with sepsis underly an acute and organism-wide hyperinflammatory response and multiple organ dysfunction; however, crosstalk between systemic metabolomic alterations and metabolic reprogramming at organ levels remains unknown. We analyzed substrate utilization by the respiratory exchange ratio, energy expenditure, metabolomic screening, and transcriptional profiling in a cecal ligation and puncture model to show that sepsis increases circulating free fatty acids and acylcarnitines but decreases levels of amino acids and carbohydrates, leading to a drastic shift in systemic fuel preference. Comparative analysis of previously published metabolomics from septic liver indicated a positive correlation with hepatic and plasma metabolites during sepsis. In particular, glycine deficiency was a common abnormality of the plasma and liver during sepsis. Interrogation of the hepatic transcriptome in septic mice suggested that the septic liver may contribute to systemic glycine deficiency by downregulating genes involved in glycine synthesis. Interestingly, intraperitoneal injection of the pyruvate dehydrogenase kinase (PDK) inhibitor dichloroacetate reversed sepsis-induced anorexia, energy imbalance, inflammation, dyslipidemia, hypoglycemia, and glycine deficiency. Collectively, our data indicated that PDK inhibition rescued systemic energy imbalance and metabolic dysfunction in sepsis partly through restoration of hepatic fuel metabolism.

## Introduction

Sepsis is a life-threatening condition associated with dysregulated host inflammatory and immune responses to an infection, resulting in immunometabolic suppression, organ failure, and high mortality (1–11). However, there is limited knowledge concerning the metabolic alterations in non–immune cells from vital organs in response to sepsis. Metabolic tissues, such as the liver and muscle, metabolically reprogram during sepsis with significant consequences for host survival (12–14), indicating systemic metabolism may be subject to dysregulation during severe infection. Therefore, it is important to understand the connection between metabolic and bioenergetic processes and their contribution to the pathogenesis and/or resolution of sepsis.

Metabolomic research in sepsis has provided an atlas of mortality-associated metabolites (15–18). However, knowledge regarding crosstalk between systemic metabolic profiles and intraorgan metabolic reprogramming remains scattered and without clarification of cause and effect in response to sepsis. Indeed, circulating carbohydrates and other small organic molecules and fatty acids are significantly altered in sepsis (19–23), although the underlying mechanisms accounting for these changes and their clinical significance are uncertain, as are their potential as therapeutic targets. Accordingly, we undertook a comprehensive metabolomic screening and energy expenditure of the consequences of sepsis in a murine model of cecal ligation and puncture (CLP) to gain insight into the systemic metabolic manifestations of chronic sepsis. Furthermore, we undertook a comparative analysis of previously published hepatic metabolomics during sepsis to shed light on the potential role of the liver in promoting the systemic metabolic phenotype of sepsis (13). Our study focused on the disease tolerance sepsis state as reported (13, 24) and targeted the mitochondrial pyruvate gate for pyruvate oxidation as a potential contributor to systemic metabolic and bioenergetic reprogramming during sepsis.

## Results

### Sepsis shifts fuel utilization and reduces systemic energy expenditure.

**Figure 1:**
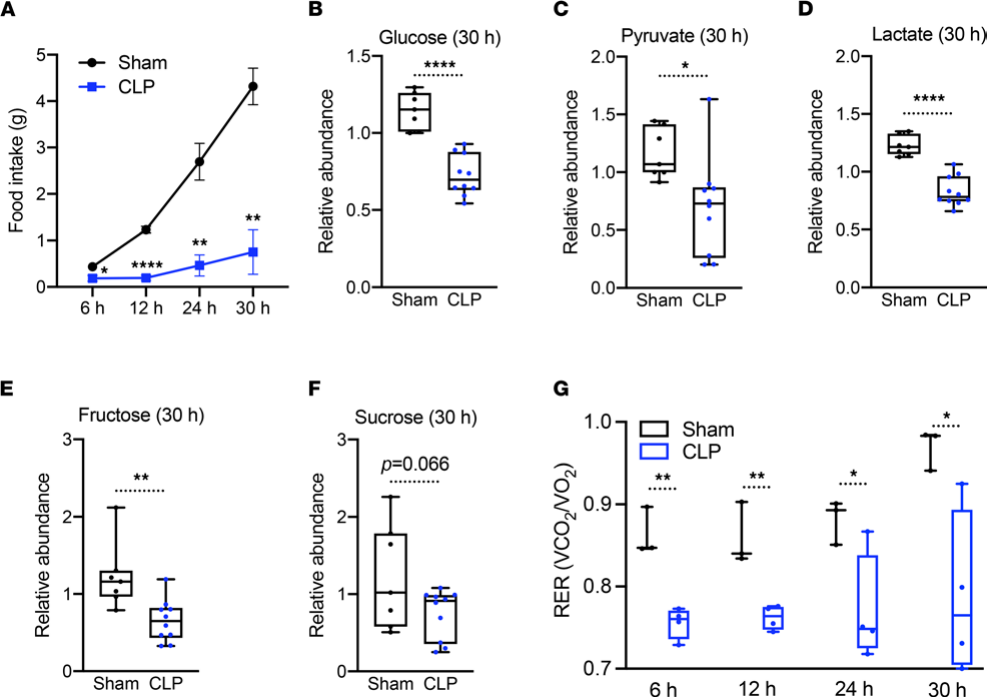
Sepsis reprograms systemic metabolism along with carbohydrate reduction. (**A**) Cumulative food intake of sham and cecal ligation and puncture (CLP) mice for 30 hours (*n* = 3 sham; 4 CLP). (**B–F**) Relative carbohydrate levels measured by UPLC-MS/MS from plasma of sham and CLP mice 30 hours after surgery (*n* = 7 sham; 10 CLP). (**G**) Respiratory exchange ratio (RER) of sham and CLP mice at various time points (*n* = 3 sham; 4 CLP). *P < 0.05, **P < 0.01, ****P < 0.0001. Statistical significance was determined using an unpaired 2-tailed Student’s *t* test.

To investigate the systemic consequences of sepsis, we first measured food intake up to 30 hours after induction of CLP using metabolic chambers. In line with previous findings (25, 26), food intake was significantly reduced in response to polymicrobial infection compared with the sham control (Figure 1A). We next performed unbiased global metabolomics on plasma of control and septic mice at 30 hours after CLP in alignment with our previous studies of the tissue tolerance phenotype of sepsis (13, 24) by ultrahigh-performance liquid chromatography–tandem mass spectroscopy (UPLC-MS/MS). Plasma levels of glucose, fructose, pyruvate, and lactate were significantly lower in septic versus control mice, while circulating sucrose levels showed a trend toward lower concentrations (*P* = 0.066) (Figure 1, B–F).

We next interrogated the respiratory exchange ratio (RER) to gain further insight into the effects of sepsis on fuel utilization (27, 28). Consistent with the parallel reduction in circulating carbohydrate concentrations, septic mice showed reduced RER values close to 0.7 (Figure 1G), suggesting a shift from carbohydrates as the primary fuel source to fatty acids, in contrast to controls, in which the RER value was closer to 0.9 (Figure 1G).

**Figure 2:**
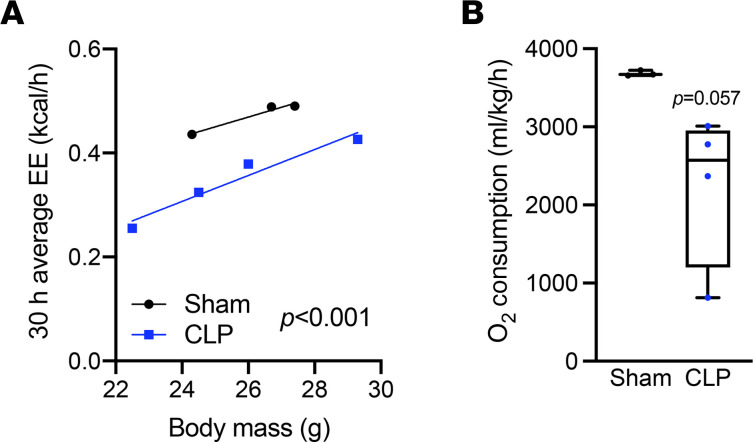
Sepsis impairs systemic energy expenditure. (**A**) Energy expenditure (EE) ANCOVA analysis assessed with body mass of sham and CLP mice (*n* = 3 sham; 4 CLP). (**B**) O_2_ consumption measured by metabolic cages for 30 hours (*n* = 3 sham; 4 CLP). Statistical significance was determined using an unpaired 2-tailed Student’s *t* test.

We next compared energy expenditure adjusted by body mass of sham and CLP mice. Septic mice showed a significant decrease in energy expenditure regardless of body weight (Figure 2A) in addition to a trend (*P* = 0.057) of less oxygen consumption (Figure 2B). These data suggest that sepsis induced a state of anorexia that was associated with a shift from carbohydrate to fat as a primary fuel source, culminating in impaired systemic metabolism.

### Sepsis dysregulates circulating lipids and amino acids.

**Figure 3:**
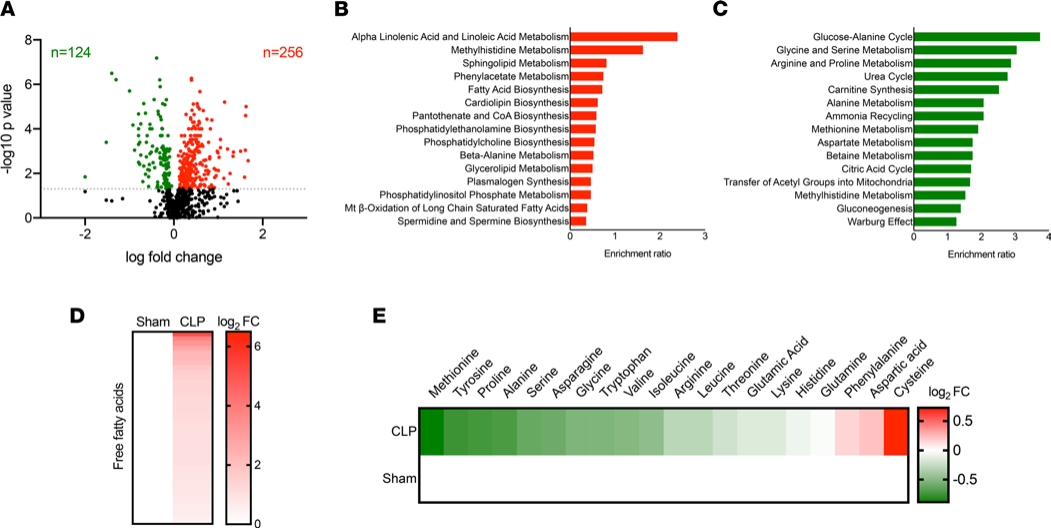
Sepsis elicits dyslipidemia and depletes circulating amino acids and carbohydrates. (**A**) Volcano plot of significantly altered plasma metabolites from sham and CLP mice measured by UPLC-MS/MS (*n* = 7 sham; 10 CLP). Statistical significance was determined using an unpaired 2-tailed Student’s *t* test. The gray dotted line indicates *P* = 0.05. (**B** and **C**) Top 15 metabolic pathways subject to accumulated metabolites (**B**) and reduced metabolites (**C**) in plasma identified by enrichment analysis of sham versus CLP mice (*n* = 7 sham; 10 CLP). (**D** and **E**) Heatmap depiction of average log_2_ fold change in plasma free fatty acids (**D**) and amino acids (**E**) in sham and CLP mice 30 hours after surgery measured by UPLC-MS/MS (*n* = 7 sham; 10 CLP).

In order to further investigate sepsis-induced changes in circulating metabolites contributing to energy imbalance, we evaluated our global unbiased metabolomic screening in plasma from sham and CLP mice. Among 782 metabolites detected, 256 metabolites significantly accumulated (red) and 124 metabolites significantly decreased (green) in septic mice (Figure 3A). We next performed enrichment analysis using small molecule pathway database (SMPDB) by MetaboAnalyst 5.0 (https://www.metaboanalyst.ca) of those metabolites significantly altered by sepsis. Pathway analysis revealed that perturbations in lipid metabolism contributed importantly to the plasma metabolomic signature of septic mice, whereas pathways of amino acid metabolism were generally depressed (Figure 3, B–E). Together, these data suggest that sepsis led to dyslipidemia and depletion of amino acids in the circulation.

### Hepatocytes contribute to systemic dyslipidemia during sepsis.

**Figure 4:**
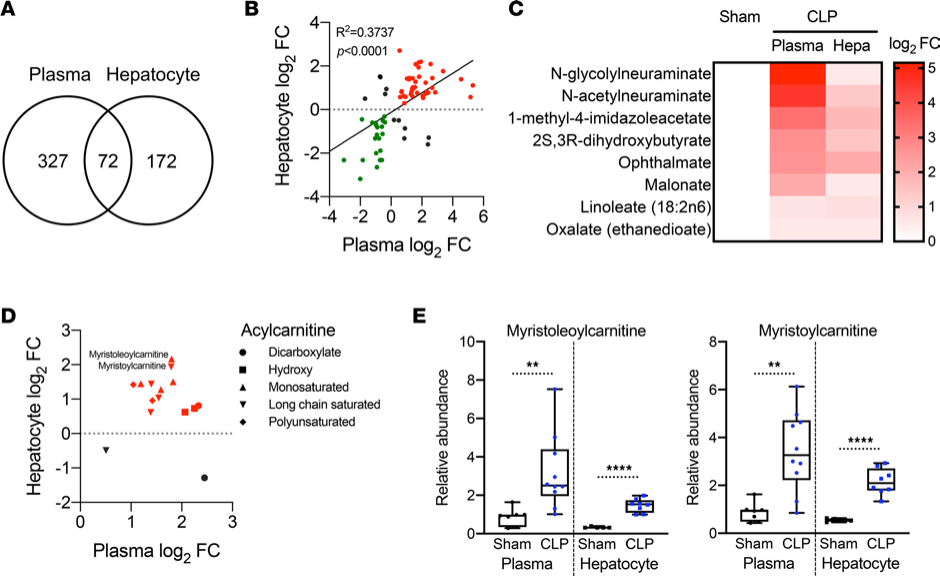
Systemic dyslipidemia correlates with hepatic steatosis during sepsis. (**A**) Venn diagram of significantly altered metabolites from plasma and isolated hepatocytes in sham and CLP mice 30 hours after surgery measured by UPLC-MS/MS (*n* = 7 sham; 10 CLP for plasma, *n* = 5 sham; 8 CLP for hepatocytes). (**B**) Correlation analysis with overlapping metabolites between plasma and isolated hepatocytes in sham and CLP mice 30 hours after surgery measured by UPLC-MS/MS (*n* = 7 sham; 10 CLP for plasma, *n* = 5 sham; 8 CLP for hepatocytes). Red and green indicate fold increases and decreases in plasma and hepatocytes, respectively. Statistical significance was determined using a simple linear regression analysis. (**C**) Heatmap depiction of average log_2_ fold change in fatty acids showing positive correlation between plasma and isolated hepatocytes in sham and CLP mice 30 hours after surgery measured by UPLC-MS/MS (*n* = 7 sham; 10 CLP). (**D**) Distribution of various acylcarnitine species plotted by average fold change in plasma and isolated hepatocytes in sham and CLP mice 30 hours after surgery measured by UPLC-MS/MS (*n* = 7 sham; 10 CLP for plasma, *n* = 5 sham; 8 CLP for hepatocytes). (**E**) Representative acylcarnitine species significantly accumulated in plasma and isolated hepatocytes in CLP mice compared with sham mice 30 hours after surgery measured by UPLC-MS/MS (*n* = 7 sham; 10 CLP for plasma, *n* = 5 sham; 8 CLP for hepatocytes). **P < 0.01, ****P < 0.0001. Statistical significance was determined using an unpaired 2-tailed Student’s *t* test.

We and others previously reported that sepsis promotes hepatic steatosis in both rodents and humans (13, 29, 30). Given that hepatocytes are involved in the dynamics of circulating free fatty acids (FFAs) (31), we sought to determine whether there is a connection between hepatic steatosis and systemic dyslipidemia in sepsis. Comparing previously published hepatic metabolomics (13) with our plasma metabolomic screen revealed that 399 plasma and 244 liver metabolites were significantly altered in experimental sepsis. Among those metabolites, 72 showed overlap between plasma and hepatocytes (Figure 4A), and this relationship was positively correlated (Figure 4B). In addition, the plasma and hepatic metabolites that accumulated in sepsis (Figure 4B) were enriched in fatty acids (Figure 4C), as previously reported in humans (22).

Acylcarnitines are reported to rewire stress-response inflammatory signaling mediators in monocytes, inducing the secretion of inflammatory cytokines and chemokines (32). Furthermore, starvation leads to increased concentrations of serum and liver acylcarnitines (33). Increases in acylcarnitine species represented another class of metabolites that accumulated in the plasma and liver (Figure 4D). Two representative acylcarnitines, myristoleoylcarnitine and myristoylcarnitine, showed significant increases in both plasma and hepatocytes (Figure 4E), consistent with the notion that hepatic and/or adipose tissue acylcarnitines may contribute to the systemic dyslipidemia of sepsis.

### Sepsis dysregulates glycine metabolism.

**Figure 5:**
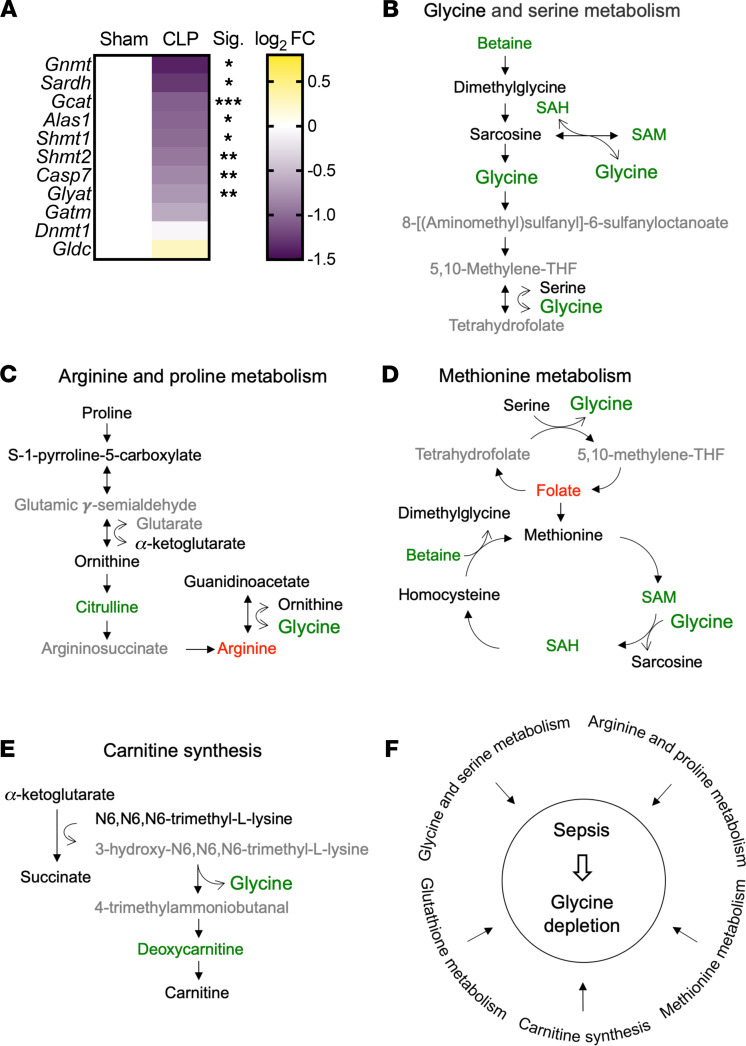
Sepsis dysregulates glycine metabolism. (**A**) Heatmap depiction of average log_2_ fold change in gene expression involved in glycine metabolism assessed by RNA-Seq in sham, CLP, and CLP + DCA 30 hours after surgery (*n* = 4 mice per group). *P < 0.05, **P < 0.01, ***P < 0.001. Statistical significance was determined using an unpaired 2-tailed Student’s *t* test. (**B–E**) Schematic representation of hepatic metabolites contributing to glycine depletion during chronic sepsis. Red denotes a metabolite increased in response to sepsis; green indicates a metabolite decreased in response to sepsis; black indicates a metabolite unchanged in response to sepsis; gray indicates a metabolite not measured in our metabolomic screening. (**F**) Metabolic pathways leading to glycine depletion during sepsis.

We previously reported that mouse septic hepatocytes have low levels of glycine (13), consistent with decreases in plasma glycine levels (Figure 3E). Glycine is synthesized from serine, threonine, choline, and hydroxyproline via interorgan metabolism, primarily by the liver and kidneys (34). In order to investigate a possible hepatic contribution to systemic glycine deficiency in sepsis, we reevaluated our previously published RNA-Seq gene expression data with regard to enzymes involved in glycine metabolism in livers 30 hours after sepsis, available in NCBI’s Gene Expression Omnibus (GEO GSE167127) (13). Eight genes involved in the regulation of glycine levels significantly decreased, including glycine N-methyltransferase (*Gnmt*), serine hydroxymethyltransferase 1 (*Shmt1*), *Shmt2*, caspase 7 (*Casp7*), sarcosine dehydrogenase (*Sardh*), glycine C-acetyltransferase (*Gcat*), 5′-aminolevulinate synthase 1 (*Alas1*), and glycine-N-acyltransferase (*Glyat*) (Figure 5A).

In line with altered transcriptional regulation of these genes, we found that sepsis changed the relative abundance of metabolites that lead to glycine deficiency. In particular, betaine, S-adenosyl-L-homocysteine (SAH), and S-adenosylmethionine (SAM) were reduced in the glycine and serine metabolic pathway (Figure 5B). GNMT reversibly converts sarcosine and SAH to glycine and SAM (35). Downregulation of *Gnmt* gene expression during sepsis was the most robust among those genes involved in glycine metabolism (Figure 5A). Sepsis also reduced hepatic citrulline, concomitant with a shift in hepatic arginine in the arginine and proline metabolic pathway (Figure 5C), suggesting glycine may be utilized as a substrate to support arginine synthesis as an alternative/additional mechanism contributing to glycine deficiency.

Folate accumulated in septic livers as a metabolite in the methionine metabolic pathway (Figure 5D). SHMT converts tetrahydrofolate and serine to 5,10-methylene-THF and glycine (36), and this component of one-carbon metabolism was significantly downregulated in septic livers (Figure 5A), possibly contributing to glycine deficiency. In contrast, metabolites in the carnitine synthetic pathway did not show distinct alterations, and SHMT also produced glycine as a side product using 3-hydroxy-N6,N6,N6-trimethyl-L-lysine and 4-trimethylammoniobutanal as a substrate and a resultant product, respectively (Figure 5E).

We also previously reported that sepsis depletes hepatic glutathione levels (13), which may be a consequence of glycine deficiency. These alterations in hepatic metabolites also aligned with changes in circulating metabolites (Supplemental Figure 1; supplemental material available online with this article; https://doi.org/10.1172/jci.insight.153944DS1). Taken together, multiple metabolic pathways that affect glycine levels were modulated by sepsis in the liver (Figure 5F).

### DCA restores feeding behavior and fuel utilization.

**Figure 6:**
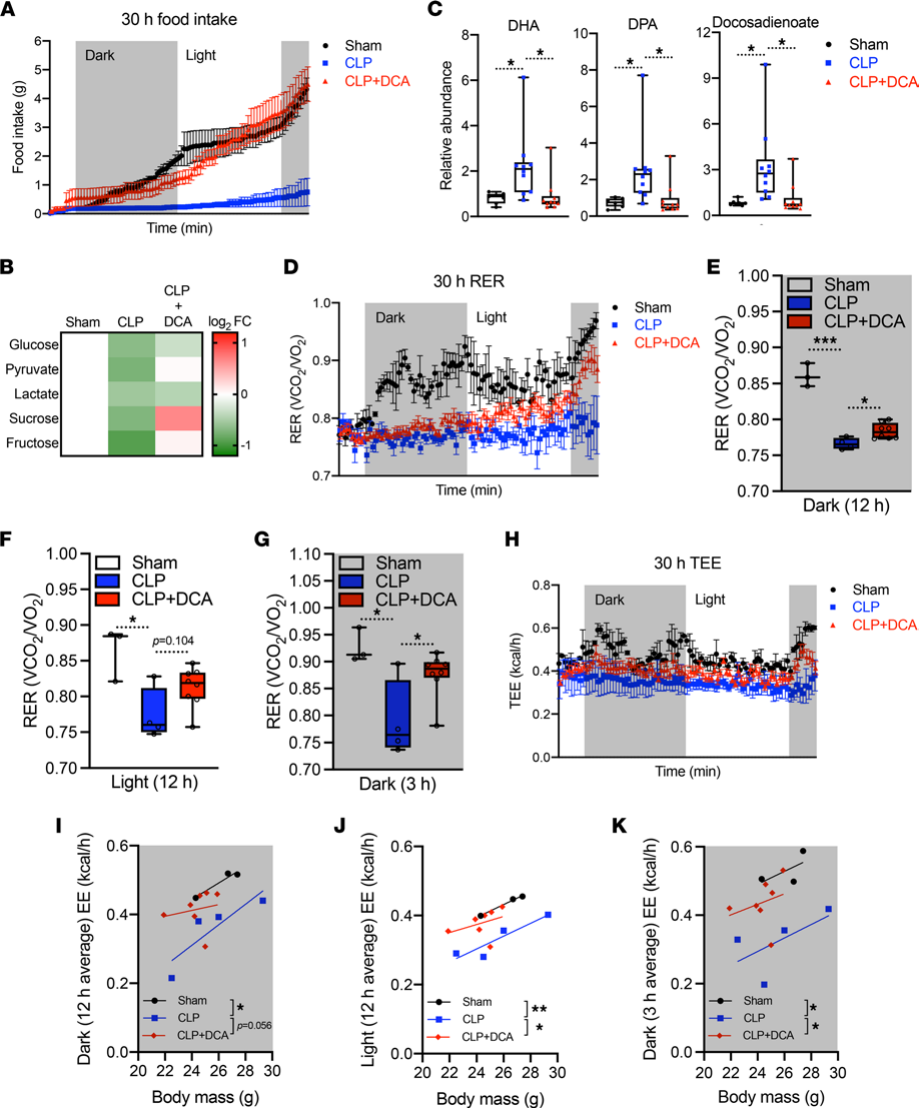
DCA recovers sepsis-induced anorexia and reprograms systemic fuel utilization. (**A**) Cumulative food intake of sham, CLP, and CLP + DCA over 30 hours (*n* = 3 sham; 4 CLP; 7 CLP + DCA). (**B**) Heatmap depiction of average log_2_ fold change in carbohydrate levels measured by UPLC-MS/MS from plasma of sham, CLP, and CLP + DCA 30 hours after surgery (*n* = 3 sham; 4 CLP; 7 CLP + DCA). (**C**) Relative fatty acid levels measured by UPLC-MS/MS from plasma of sham, CLP, and CLP + DCA 30 hours after surgery (*n* = 7 sham; 10 CLP; 10 CLP + DCA). (**D**) RER of sham, CLP, and CLP + DCA for 30 hours (*n* = 3 sham; 4 CLP; 7 CLP + DCA). Average RER for (**E**) 12-hour dark cycle, (**F**) 12-hour light cycle, and (**G**) 3-hour dark cycle. (**H**) Total energy expenditure (TEE) of sham, CLP, and CLP + DCA for 30 hours (*n* = 3 sham; 4 CLP; 7 CLP + DCA). Average EE for (**I**) 12-hour dark cycle, (**J**) 12-hour light cycle, and (**K**) 3-hour dark cycle. *P < 0.05, **P < 0.01, ***P < 0.001. Statistical significance was determined using 1-way ANOVA.

We have previously reported that pyruvate dehydrogenase kinase (PDK) inhibition by dichloroacetate (DCA) rebalances immunometabolic and mitochondrial respiration and anabolic energetics during sepsis, thereby increasing survival in septic mice (24). Furthermore, DCA reverses dysregulated hepatocyte metabolism, including triglyceride accumulation and mitochondrial dysfunction (13). Given that DCA restores the hepatic metabolome and increases hepatic mitochondrial energetics to promote survival in septic mice, we hypothesized that PDK inhibition might also reverse sepsis-induced anorexia. To assess this, we housed septic mice treated with or without DCA in metabolic cages after sepsis onset. Strikingly, DCA administration completely normalized food intake in CLP mice when administered 1 hour after CLP (Figure 6A). DCA alone did not have effects on food intake stimulation in normal mice (Supplemental Figure 3). DCA treatment also rebalanced abnormal concentrations of circulating carbohydrates (Figure 6B) and FFAs (Figure 6C). Together, these data support the notion that stimulating pyruvate dehydrogenase megacomplex by reducing function of its principal inactivator, PDK, may restore systemic fuel availability during sepsis.

To confirm that metabolite rewiring might lead to changes in systemic energetics, we assessed DCA’s effects on RER and energy expenditure. Decreased RER in septic mice was reversed by DCA in a time-dependent manner throughout the 30 hours after CLP (Figure 6D). Of note, RER in the dark (feeding) cycles showed significant recovery by DCA (Figure 6, E and G) with a modest trend (*P* = 0.104) during the light cycle (Figure 6F), compatible with circadian rhythm potentially affecting sepsis mortality (37). Reduced total energy expenditure was also rescued by DCA during sepsis (Figure 6H). ANCOVA analysis assessed with body mass showed that the 12 hours of average energy expenditure in the first dark cycle elicited a strong trend (*P* = 0.056) of restoration by DCA during sepsis (Figure 6I). The same analysis in the light cycle and the second dark cycle exhibited significant restoration by DCA during sepsis (Figure 6, J and K). Collectively, these data suggest that PDK inhibition ameliorated sepsis by decreasing anorexia and reprograming systemic fuel utilization.

### DCA ameliorates acylcarnitine accumulation during sepsis.

**Figure 7:**
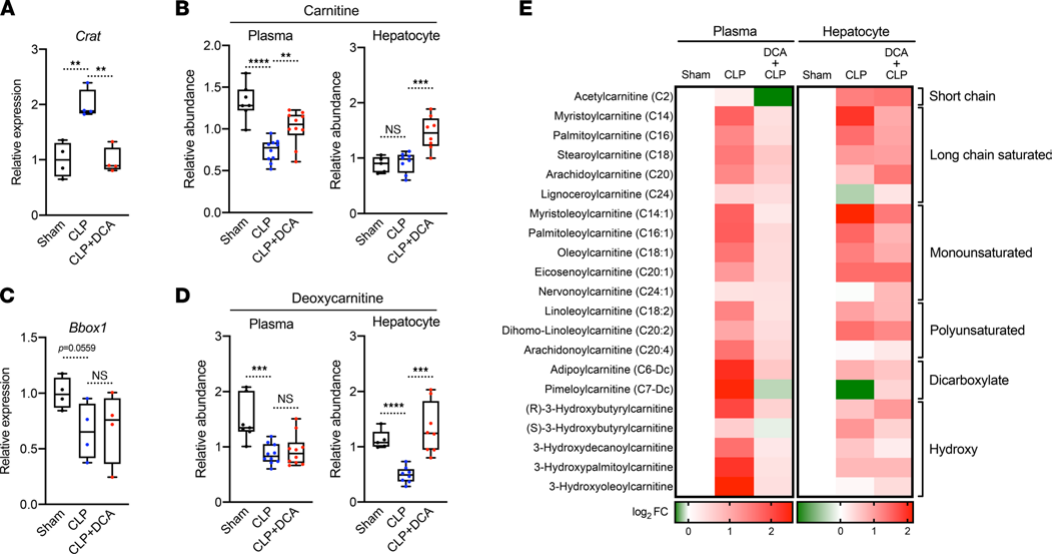
DCA reduces acylcarnitines during sepsis. (**A**) Relative gene expression of carnitine acyltransferase (*Crat*) assessed by RNA-Seq in sham, CLP, and CLP + DCA 30 hours after surgery (*n* = 4 mice per group). (**B**) Relative carnitine levels measured by UPLC-MS/MS from plasma of sham, CLP, and CLP + DCA 30 hours after surgery (*n* = 7 sham; 10 CLP; 10 CLP + DCA). (**C**) Relative gene expression of *Bbox1* assessed by RNA-Seq in sham, CLP, and CLP + DCA 30 hours after surgery (*n* = 4 mice per group). (**D**) Relative deoxycarnitine levels measured by UPLC-MS/MS from plasma of sham, CLP, and CLP + DCA 30 hours after surgery (*n* = 7 sham; 10 CLP; 10 CLP + DCA). (**E**) Heatmap depiction of average log_2_ fold change in acylcarnitine levels measured by UPLC-MS/MS from plasma of sham, CLP, and CLP + DCA 30 hours after surgery (*n* = 3 sham; 4 CLP; 7 CLP + DCA). **P < 0.01, ***P < 0.001, ****P < 0.0001. Statistical significance was determined using 1-way ANOVA.

Given that DCA may reverse the switch from carbohydrate to FFA utilization during sepsis, we next evaluated its effects on acylation of carnitines. This was done in consideration of their role in balancing sugar and lipid metabolism (38) and of the liver being a major contributor to systemic acylcarnitine levels (39, 40). Accordingly, we found that hepatic expression of carnitine acyltransferase (*Crat)* in septic mice was significantly upregulated and reversed by DCA (Figure 7A). In accordance with altered *Crat* expression, carnitine levels in the circulation were significantly reduced during sepsis, and this reduction was also restored by DCA (Figure 7B). However, hepatic carnitine levels remained unaltered during sepsis, possibly due to compensatory biosynthesis (Figure 7B). The rate-limiting function of gamma-butyrobetaine dioxygenase (BBOX1) catalyzes mature carnitine from its precursor deoxycarnitine. *Bbox1* mRNA levels showed a decreasing trend (*P* = 0.0559) in septic livers (Figure 7C), and both circulating and hepatic deoxycarnitine levels significantly decreased in septic mice. DCA restored only hepatic deoxycarnitine levels (Figure 7D). Considering that deoxycarnitine is converted to carnitine by BBOX1, hyperactivation of BBOX1 could occur and contribute to high flux of conversion from deoxycarnitine to carnitine in the septic liver. As *Crat* expression implied, sepsis led to acylcarnitine accumulation in hepatocytes as well as in the circulation, and DCA reversed this phenomenon (Figure 7E). In sum, these data suggest that DCA normalized excessive acylcarnitine contents in the circulation and liver, regulating hepatic gene expression involved in acylation of carnitines.

### DCA reinstates sepsis-induced glycine deficiency.

**Figure 8:**
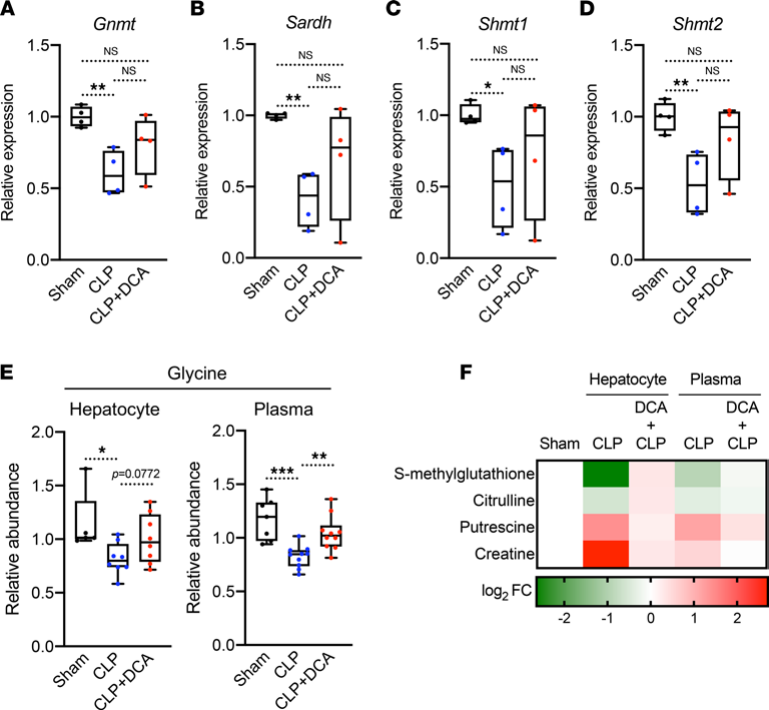
DCA reinstates sepsis-induced metabolic alterations shared by the liver and plasma. (**A–D**) Relative gene expression involved in glycine metabolism assessed by RNA-Seq in sham, CLP, and CLP + DCA 30 hours after surgery (*n* = 4 mice per group). (**E**) Relative glycine levels measured by UPLC-MS/MS from plasma of sham, CLP, and CLP + DCA 30 hours after surgery (*n* = 7 sham; 10 CLP; 10 CLP + DCA). (**F**) Heatmap depiction of average log_2_ fold change in glycine pathway metabolites measured by UPLC-MS/MS from plasma of sham, CLP, and CLP + DCA 30 hours after surgery (*n* = 3 sham; 4 CLP; 7 CLP + DCA). *P < 0.05, **P < 0.01, ***P < 0.001. Statistical significance was determined using 1-way ANOVA.

Given that sepsis dysregulates glycine metabolism in mice, we examined whether DCA might blunt glycine deficiency during sepsis by assessing *GNMT, SARDH, SHMT1,* and *SHMT2*, the key enzymes involved in glycine metabolism (Supplemental Figure 2). Significant downregulation of these hepatic genes during sepsis reversed after DCA administration (Figure 8, A–D). DCA treatment resulted in a trend (*P* = 0.077) for reversed glycine levels by DCA in the septic liver and a significant recovery in circulating glycine levels (Figure 8E). DCA treatment of septic mice also reversed sepsis-regulated metabolites in the glycine pathway in the plasma and liver (Figure 8F). These metabolites included S-methyl glutathione (glutathione metabolism), citrulline (arginine and proline metabolism), putrescine (methionine metabolism), and creatine (glycine and serine metabolism), implicating them as liver-driven metabolites that might contribute to systemic glycine deficiency during sepsis.

### DCA reduces inflammation and morbidity regardless of food restriction during sepsis.

**Figure 9:**
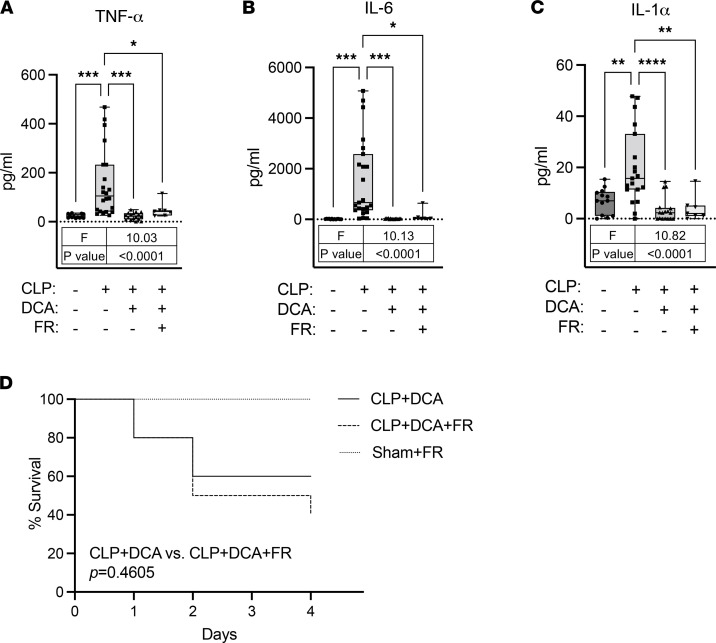
DCA reverses activation of inflammatory cytokines and morbidity regardless of food restriction during sepsis. (**A–C**) Inflammatory cytokines measured by ELISA from plasma of sham, CLP, CLP + DCA, and CLP + DCA + food restriction (FR) 30 hours after surgery (*n* = 13 sham; 19–23 CLP; 15–17 CLP + DCA; 7 CLP + DCA + FR). Values outside of lower outlier gate and upper outlier gate were removed from analysis (See *Statistics* for details). The number of outliers was as follows: **A** (1 CLP; 2 CLP + DCA; 2 CLP + DCA + FR), **B** (4 CLP + DCA; 2 CLP + DCA + FR), and **C** (4 CLP; 2 CLP + DCA; 2 CLP + DCA + FR). (**D**) Kaplan-Maier survival curve showing similar survival rate (Mantel-Cox log-rank test: *P* = 0.4605) between CLP + DCA and CLP + DCA + FR (*n* = 10 in each of 3 cohorts). *P < 0.05, **P < 0.01, ***P < 0.001, ****P < 0.0001. Statistical significance was determined using 1-way ANOVA.

**Figure 10:**
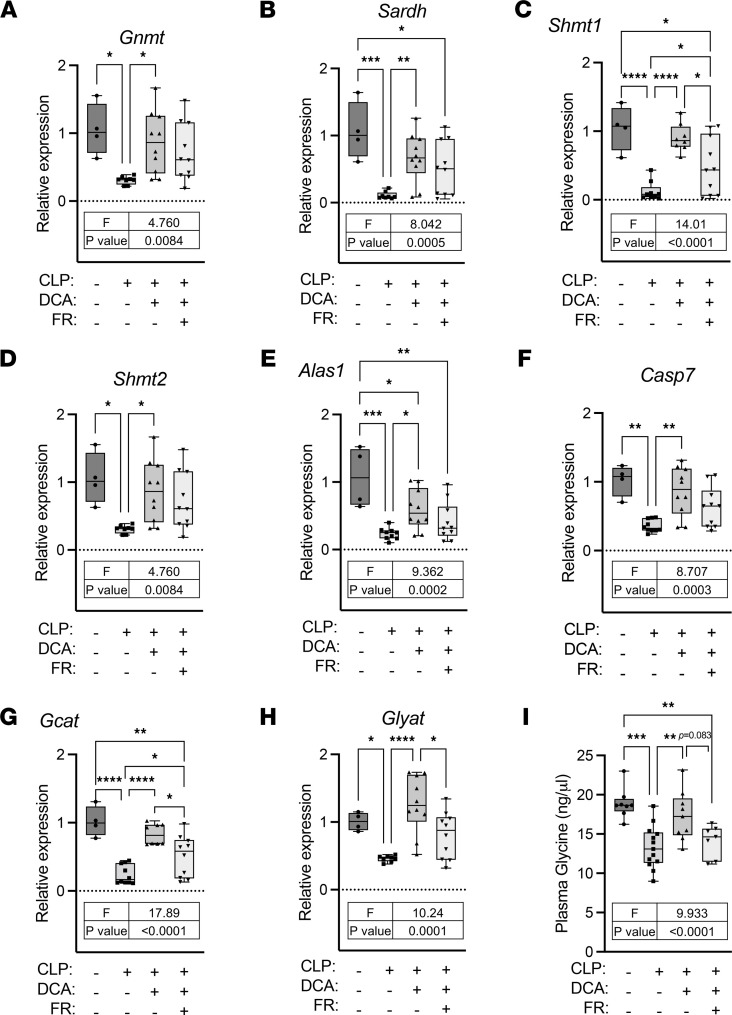
Food restriction blunts effects of DCA on restoration of glycine metabolism. (**A–H**) Relative gene expression involved in glycine metabolism assessed by qRT-PCR from livers of sham, CLP, CLP + DCA, and CLP + DCA + food restriction (FR) 30 hours after surgery (*n* = 4 sham; 8–10 CLP; 8–10 CLP + DCA; 10 CLP + DCA + FR). (**I**) Glycine levels were measured by glycine assay kits from plasma of sham, CLP, CLP + DCA, and CLP + DCA + FR 30 hours after surgery (*n* = 8 sham; 13 CLP; 9 CLP + DCA; 7 CLP + DCA + FR). Values outside of lower outlier gate and upper outlier gate were removed from analysis (See *Statistics* for details). The number of outliers was as follows: **A** (2 CLP), **B** (2 CLP), **C** (1 CLP; 2 CLP + DCA), **D** (2 CLP), **E** (1 CLP), **G** (2 CLP + DCA), **H** (2 CLP), and **I** (1 sham; 2 CLP + DCA + FR). *P < 0.05, **P < 0.01, ***P < 0.001, ****P < 0.0001. Statistical significance was determined using 1-way ANOVA.

Considering DCA restores feeding behavior during sepsis, we set up to investigate whether food consumption in DCA-treated septic mice governs amelioration of sepsis morbidity. It has been shown that proinflammatory cytokines are elevated in the early stage of inflammation in a CLP septic mouse model (41). We tested whether DCA reduces inflammation of septic mice in the context of food restriction, which allows only 1 g of food daily (based on average daily food consumption of CLP mice (Figure 6A). As expected, the levels of TNF-α, IL-6, and IL-1α were significantly increased in response to CLP (Figure 9, A–C). Interestingly, DCA normalized all the inflammatory cytokines irrespective of food restriction (Figure 9, A–C). Next, we assessed the effects of DCA and food restriction in CLP mice on survival. Since food restriction in sham mice displayed severe lethargic behavior with 25.2% ± 2.3% of body weight loss on day 5, we analyzed survival up to day 4. A Kaplan-Maier survival curve showed that food restriction did not alter survival rates (Mantel-Cox log-rank test: *P* = 0.4605) in DCA-treated CLP mice (Figure 9D). This suggests that by day 4, survival with DCA did not require restoration of feeding behavior, given the survival rates were similar. However, hepatic gene expression showed that food restriction blunted DCA effects on restoration of glycine metabolism (Figure 10, A–H). In line with this, plasma glycine levels showed a decreasing trend (*P* = 0.083) in response to food restriction in DCA-treated CLP mice (Figure 10I), suggesting that DCA recuperated, at least in part, circulating plasma metabolites via the restoration of food intake. Taken together, these data indicate that DCA ameliorated inflammation and morbidity in septic mice independently of feeding behavior but restored circulating metabolites partly by reversal of anorexia.

## Discussion

**Figure 11:**
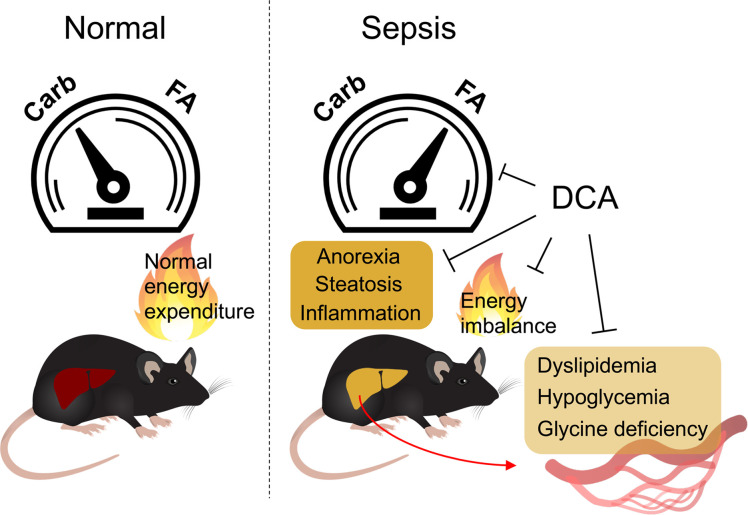
DCA rescues deleterious metabolic alterations during sepsis. Schematic diagram describing detrimental alterations in response to sepsis and reversion effects of DCA.

Energy metabolism is disrupted during sepsis and is a major contributor to organismal demise (42, 43). Better understanding of interaction and dynamics between systemic metabolic alterations and multiorgan failure provides a logical foundation for therapeutic interventions in sepsis (44). In the present study, we found that sepsis elicited inflammation and anorexia, altered fuel availability/utilization, and ultimately, culminated in systemic energy imbalance in mice. We identified a possible hepatic contribution of sepsis-induced dyslipidemia and glycine deficiency as a potential target. Our data indicated that administration of the PDK inhibitor DCA reversed dysregulated systemic energy balance and metabolic dysfunction partly through hepatic metabolic regulation, which may ultimately underlie the protective effects of DCA during sepsis (Figure 11).

Fuel utilization during sepsis has been addressed for several decades given its role in governing alterations of systemic metabolism in critical illness (45–48). Fluctuations in circulating glucose occur commonly in sepsis, and hypoglycemia has been inversely associated with survival (47–49). The murine CLP model also been associated with hypoglycemia in the chronic phase of sepsis (13), which we also observed in the present study. Furthermore, we observed significant increases in circulating FFAs during sepsis, which may ultimately underlie the shift in fuel preference from carbohydrate sources to utilization of FFAs.

Our metabolomic analysis revealed significant reductions in the circulating levels of carbohydrates and amino acids in contrast to fatty acids. Others have reported that patients with sepsis have reduced plasma concentrations of most amino acids, and higher infusion rates of amino acids are required to maintain plasma concentrations due to increased hepatic extraction of amino acids from plasma (19). In the present study, we found that glycine deficiency was a common abnormality of the plasma and liver during sepsis, with downregulation of hepatic gene expression for glycine synthesis. Considering glycine as a precursor of glutathione and carnitine, glycine deficiency can lead to organ damage from excessive oxidative stress. For example, glycine and cysteine supplementation in patients infected with HIV restored glutathione synthesis and mitochondrial fuel oxidation (49). Another study suggests that prefeeding of glycine reduces liver damage and dysregulated systemic inflammatory responses in a rat sepsis model (50), but significant gaps exist in our understanding of the putative beneficial effects of glycine during sepsis, and additional studies are needed. A potential caveat to interpreting the effect of DCA on glycine levels is that the drug is metabolized to glycine in humans and rodents (51), although the quantitative significance of a single dose of DCA as a contributor to circulating levels of this amino acid under the conditions reported here is likely to have little impact on total intrahepatic or circulating glycine levels.

The carnitine pool, composed of L-carnitine and its acylated derivatives, is consistently recognized as a prognostic indicator of severe sepsis and septic shock. Increased plasma acylcarnitine predicts high mortality of patients with sepsis (22, 52, 53). In line with these studies, our data suggest a hepatic contribution to systemic acylcarnitine accumulation during sepsis. We showed that hepatic *Crat* was highly expressed during sepsis, suggesting that increased acylation of carnitine in the liver contributes to elevated levels of acylcarnitines in plasma. CRAT can drive either a forward acylcarnitine synthesis and its accumulation or reverse it and increase acetyl-CoA regeneration to fuel mitochondrial energetics (54). L-carnitine supplementation in patients with septic shock elevates serum acylcarnitine levels (55), indicating that acylcarnitine synthesis is active during sepsis.

It is inevitable to consider that metabolic alterations come at least partly from the fasted or near-fasted state during sepsis. Circulating acylcarnitine and FFA levels increase during acute energetic stress, such as fasting (33, 56, 57). This encourages mining sepsis-exclusive changes in metabolites as biomarkers distinct from normal fasting outcomes. For instance, although α linolenic acid and linoleic acid show no significant alterations in response to 48 hours of fasting in mice (57), the present study highlights accumulation of metabolites involved in α linolenic acid and linoleic acid metabolism as a top enriched pathway in septic mice, concordant with results in age-matched (young) mice 12 hours after CLP in a recent study (58). Stephen et al. conducted NMR-based metabolomics in serum of CLP mice at 24 hours and used a sham with 24 hours fasting as a control. They revealed that lactate and metabolites associated with ketogenesis and branched-chain amino acid metabolism have lower abundance with increased inflammatory cytokines in the CLP group (59). It has also been reported that glucose levels are lower in CLP mice compared with a fasted sham control at 24 hours (60). These studies are consistent with our results, and DCA reversed deleterious metabolic alterations and inflammation. However, metabolic abnormalities existed in the food-restricted DCA-treated septic mice, suggesting not all but some of the metabolic alterations were attributable to food restriction. We conclude that the fasting state is indeed responsible for metabolic rewiring in septic mice, and that this is somewhat uncoupled to inflammation. Thus, further investigation on how DCA reverses sepsis-induced anorexia and inflammation is warranted.

The mitochondrial pyruvate dehydrogenase megacomplex catalyzes the rate-determining step in the aerobic oxidation of glucose and of the glycolytic product pyruvate to acetyl CoA. Rapid regulation of the complex is mediated mainly by reversible phosphorylation by any 1 of 4 PDK isoforms and 2 pyruvate dehydrogenase phosphatase isoforms (61). Pathological upregulation of PDKs, resulting in inhibition of pyruvate dehydrogenase megacomplex, has been reported in diverse acquired diseases of metabolic integration and immune dysfunction. We reported that the pan-PDK inhibitor DCA reduces sepsis mortality (24), regulates anabolic and catabolic energy supply in immune cells and hepatocytes (13, 62), and reduces TCA cycle tolerance mediator itaconate. Relevant to this is that DCA reverses hepatic metabolic dysfunction and the mitochondrial low-energy state in septic mice (13). In the present study, we showed that PDK inhibition reversed sepsis-induced anorexia, restored carbohydrate fuel metabolism, and rebalanced the carnitine/acylcarnitine ratio. Nevertheless, the majority of carnitines are stored in the heart and skeletal muscle (63), and their contribution to carnitine dynamics in the liver needs to be considered in future studies of the metabolic consequences of sepsis. Furthermore, in humans, BBOX1, the essential enzyme for carnitine synthesis, is located in the kidneys and the brain as well as in the liver (64). Therefore, investigating carnitine metabolism among these tissues in response to sepsis would fill an important knowledge gap and might inform one way that DCA improves sepsis survival in mice.

In conclusion, we showed that CLP-induced murine sepsis caused major disruption of carbohydrate, fat, and amino acid metabolism in the liver and plasma, resulting in significant perturbation of systemic bioenergetics. Pharmacological targeting of the pyruvate dehydrogenase megacomplex/PDK axis in the liver has the potential to overcome sepsis-induced hepatic immunometabolic dysfunction that leads to systemic energy crisis and thus offers a potentially new approach to the development of precision therapeutics for severe sepsis.

## Methods

### Animal experiments.

Animal experiments were conducted as previously described (13). Male C57BL/6J mice aged 8–10 weeks were purchased from The Jackson Laboratory (stock number 000664). All animals were subjected to a 12-hour light/12-hour dark cycle with ad libitum access to standard rodent chow and water except for food restriction, which allowed 1 g of chow daily. For CLP surgery, the cecum was ligated and punctured 2 times with a 22-gauge needle. The contents were then returned, and the incision was closed in 2 layers (peritoneum and skin). A sham operation where an abdominal incision was made but the cecum not ligated or punctured was used as a control. Each animal got subcutaneous fluids (1 mL normal saline) and was kept warm until fully awake. DCA (347795, Sigma-Aldrich) was administered (25 mg/kg) intraperitoneally at various time points after surgery: for metabolomic screening, RNA-Seq, and ELISA, DCA was administered 24 hours after surgery and tissues and plasma were collected 6 hours after DCA administration (30 hours after surgery); for metabolic cages and survival, DCA was administered immediately after surgery.

### Indirect calorimetry.

To measure whole body energy expenditure in live animals, mice were housed individually in metabolic chambers of PhenoMaster (indirect calorimetry system; TSE Systems) and acclimatized for 3 days with free access to food and water. The energy expenditure, oxygen consumption (VO_2_), RER, and food intake were obtained continuously during a 12-hour light/12-hour dark cycle for 30 hours (from 3:00 PM on day 1 to 9:00 PM on day 2) after surgery and DCA or vehicle administration.

### UPLC-MS/MS.

Metabolomic screening was performed by a previously described manner (13) with plasma samples from mice described above. Enrichment analysis was performed with SMPDB by MetaboAnalyst 5.0 (https://www.metaboanalyst.ca). Significantly altered metabolites by sepsis were recognized with a human metabolome database (HMDB) ID and used for overrepresentation analysis. The enrichment ratio was computed based on observed hits divided by expected hits in the given pathways.

### Inflammatory cytokine assay.

Mouse TNF-α, IL-6, and IL-1α cytokines were measured in mouse plasma using mouse DuoSet ELISA kits (DY410-05, DY406-05, DY400-05, R&D Systems) combined with DuoSet Ancillary reagent kit 2 (DY008, R&D Systems) according to the manufacturer’s instructions. Briefly, capture antibodies were diluted to working concentration with ELISA plate-coating buffer indicated in the certificate of analysis and incubated overnight at room temperature. Plasma samples were diluted at a 1:3 ratio in the reagent diluent and applied for further assay.

### RNA isolation and real-time qPCR.

RNA isolation was performed using the commercially available Aurum RNA miniprep kit (732-6820, Bio-Rad) followed by the TRIzol/chloroform method. Liver tissues were homogenized in 1 mL of TRIzol and 200 μL of chloroform was added. After mixing thoroughly, tubes were left for 5 minutes and then centrifuged for 5 minutes at full speed. The aqueous phases were mixed with 50% ethanol and subjected to the RNA binding columns. Gene expression analysis was conducted with 50 ng RNA using iTaq Universal One-Step Real-Time qPCR kit (1725140, Bio-Rad). The reaction was carried out according to the manufacturer’s instructions using CFX Connect Real-Time PCR Detection system (1855200, Bio-Rad). Probes used for TaqMan gene expression assays (Thermo Fisher Scientific) were as follows: *Alas1* (Mm01235914_m1), *Casp7* (Mm00432322_m1), *Gcat* (Mm00496962_m1), *Glyat* (Mm01195742_m1), *Sardh* (Mm00454657_m1), *Shmt1* (Mm07296291_g1), and *Shmt2* (Mm00659512_g1).

### Glycine assay.

Glycine levels were measured in plasma using a glycine assay kit (MAK261, Sigma-Aldrich) according to the manufacturer’s instructions. Plasma samples were diluted at a 1:30 ratio in the glycine assay buffer and further used for measurement.

### Statistics.

A 2-tailed unpaired *t* test was performed to determine significant relationships between groups in the experiments. One-way ANOVA was used when comparing more than 2 groups, followed by Tukey’s multiple-comparison test. Statistical analysis was performed using GraphPad Prism 9. When indicated, outliers outside of the gates were removed according to the IQR measurement: Q1 – 1.5 × IQR (lower outlier gate) and Q3 + 1.5 × IQR (upper outlier gate). Data are represented as mean ± SEM or box plots. *P* values of less than 0.05 were considered significant.

### Study approval.

All protocols and experimental procedures were reviewed and approved by the IACUC of Wake Forest School of Medicine.

## Author contributions

TSO planned and executed the study, analyzed data, and drafted the manuscript. MZ planned and executed the study and analyzed data. SJ executed the study and analyzed data. DL executed the study and analyzed data. PWS analyzed data and drafted the manuscript. CEM planned the study, analyzed data, drafted the manuscript, and supervised the project. MAQ planned and executed the study, analyzed data, drafted the manuscript, and supervised the project. All authors contributed to the article and approved the submitted version.

## Supplementary Material

Supplemental data

## Version 1 06/22/2022

Electronic publication
