# Supplementary material for: Dichloroacetate improves systemic energy balance and feeding behavior during sepsis
Source: JCI Insight. 2022 Jun 22;7(12):e153944. doi: 10.1172/jci.insight.153944 (PMC9309051; doi:10.1172/jci.insight.153944)
Supplement: Supplemental data [file jciinsight-7-153944-s248.pdf]

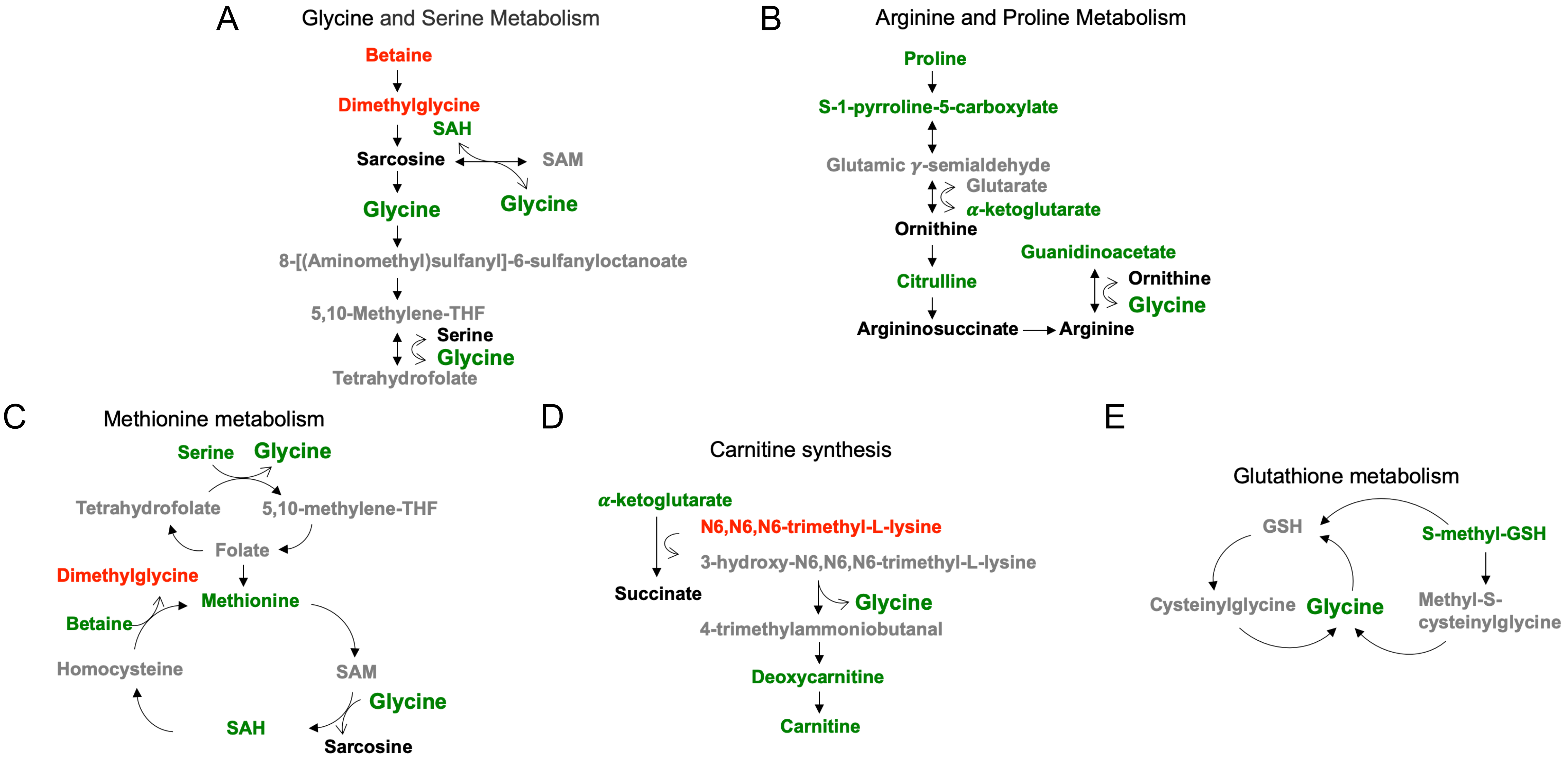

Fig. S1. Changes in circulating metabolites involved in glycine deficiency in response to sepsis. *(A-E)* Schematic representation of plasma metabolites contributing to glycine depletion during chronic sepsis. Red denotes a metabolite increased in response to sepsis; green indicates a metabolite decreased in response to sepsis; black indicates a metabolite unchanged in response to sepsis; grey indicates a metabolite not measured in our metabolomic screening.

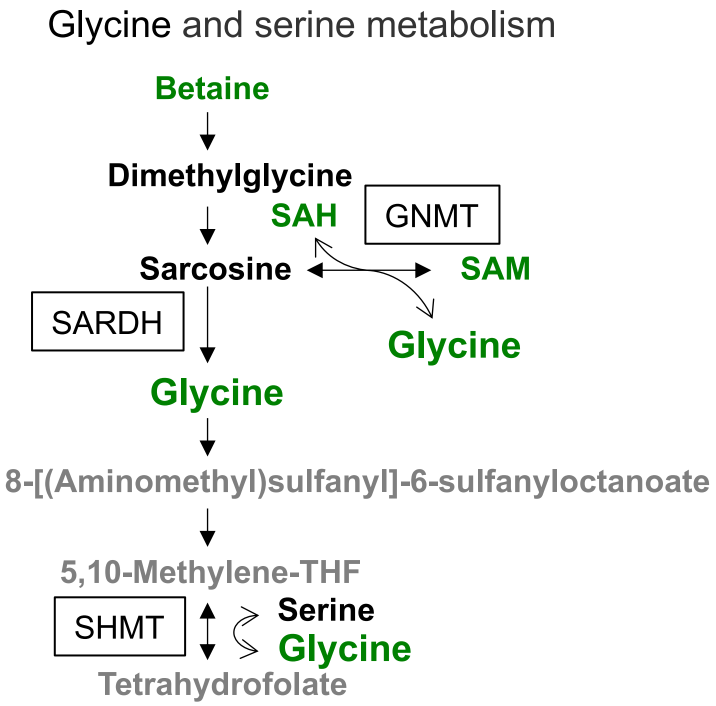

Fig. S2. Enzymes that lead to glycine deficiency during sepsis. Enzymes involved in glycine synthesis are shown in rectangle in the glycine and serine metabolic pathway. Green indicates a metabolite decreased in response to sepsis; black indicates a metabolite unchanged in response to sepsis; grey indicates a metabolite not measured in our metabolomic screening.

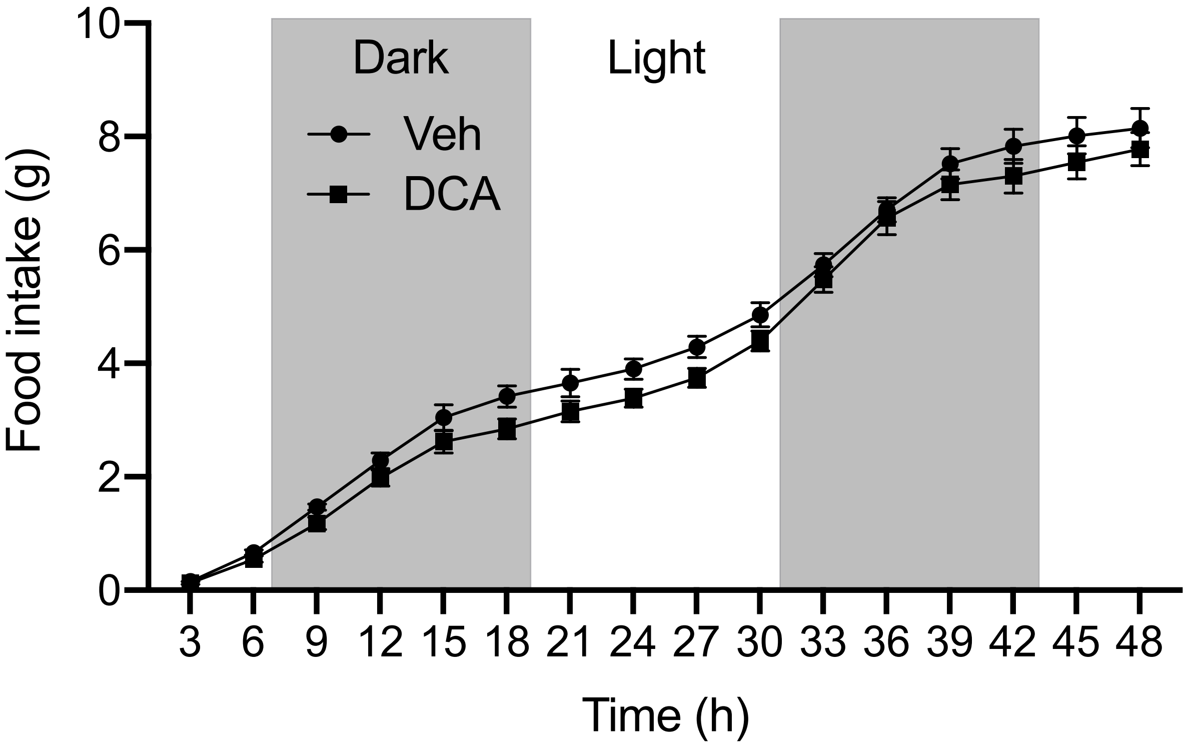

Fig. S3. Food intake after DCA administration. Mice were individually housed and food intake was measured every 3 h for 2 days after intraperitoneal administration of vehicle (Veh) or dichloroacetate (DCA). n = 5 mice per group.

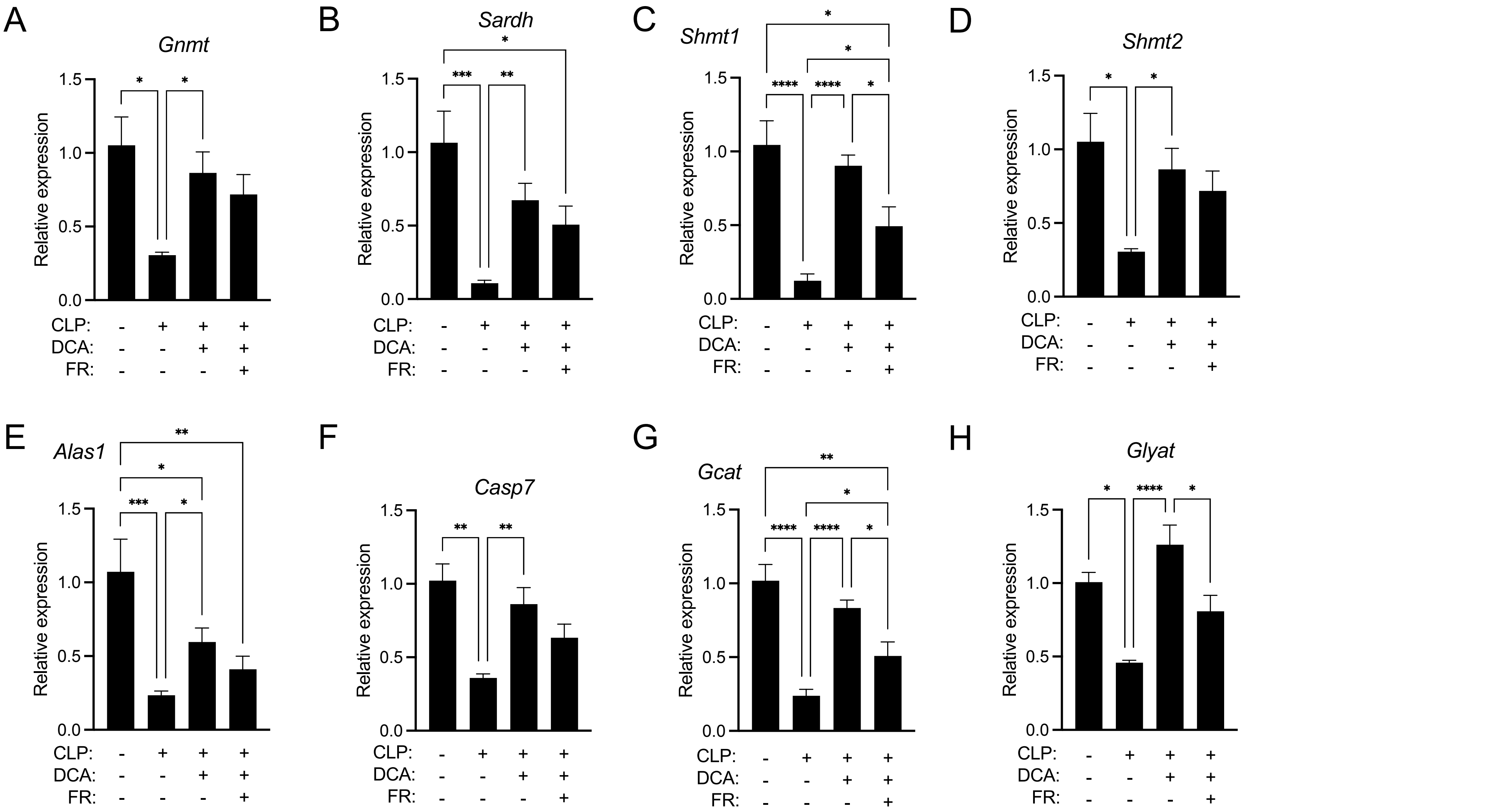

Fig. S4. Hepatic gene expression involved in glycine metabolism in response to CLP, DCA and food restriction. (*A-H*) Relative gene expression involved in glycine metabolism assessed by qRT-PCR from livers of sham, CLP, CLP+DCA, and CLP+DCA+Food restriction (FR) 30 h post-surgery (n = 4 sham; 8-10 CLP; 8-10 CLP+DCA; 10 CLP+DCA+FR). *$p < 0.05$, **$p < 0.01$, ***$p < 0.001$, ****$p < 0.0001$.

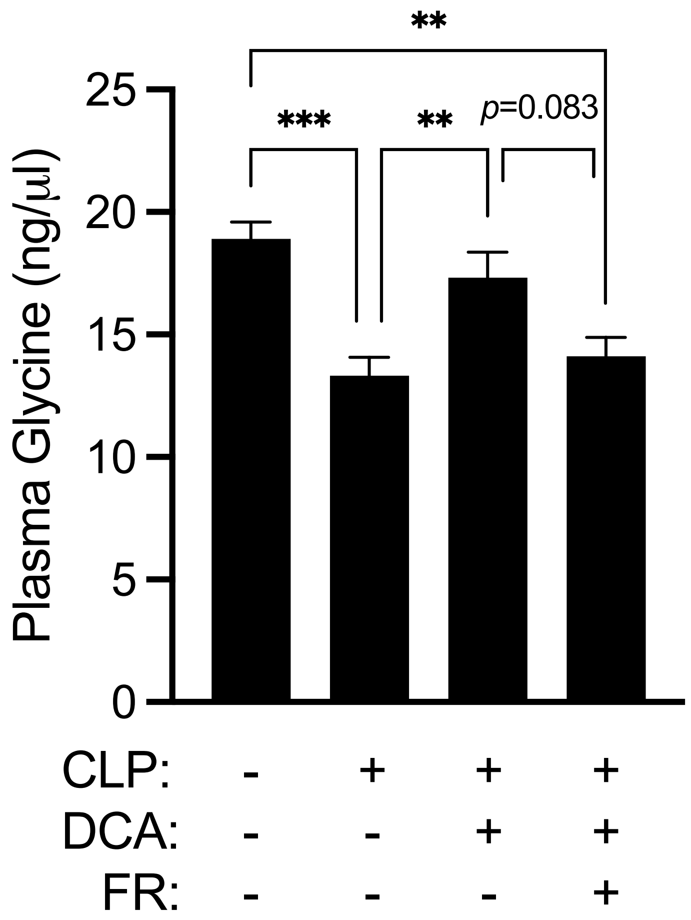

Fig. S5. Alterations in circulating glycine levels in response to CLP, DCA and food restriction. Glycine levels were measured by glycine assay kits from plasma of sham, CLP, CLP+DCA, and CLP+DCA+Food restriction (FR) 30 h post-surgery (n = 8 sham; 13 CLP; 9 CLP+DCA; 7 CLP+DCA+FR). Values outside of lower outlier gate and upper outlier gate were removed from analysis (See Statistics for detail). **$p < 0.01$, ***$p < 0.001$.
